# Flotillin-2 promotes metastasis of nasopharyngeal carcinoma by activating NF-κB and PI3K/Akt3 signaling pathways

**DOI:** 10.1038/srep11614

**Published:** 2015-07-24

**Authors:** Jie Liu, Wei Huang, Caiping Ren, Qiuyuan Wen, Weidong Liu, Xuyu Yang, Lei Wang, Bin Zhu, Liang Zeng, Xiangling Feng, Chang Zhang, Huan Chen, Wei Jia, Lihua Zhang, Xiaomeng Xia, Yuxiang Chen

**Affiliations:** 1 Cancer Research Institute, Collaborative Innovation Center for Cancer Medicine, Key Laboratory for Carcinogenesis of Chinese Ministry of Health, School of Basic Medical Science, Central South University, Xiangya Road 110, 410078, Changsha, Hunan, P. R. China; 2 Department of Pathology, Hunan Cancer Hospital, Changsha, Hunan, P. R. China; 3 Department of Gynaecology and Obstetrics, The Second Xiangya Hospital, Central South University, Changsha, Hunan, P. R. China; 4 Hepatobiliary & Enteric Surgery Research Center, Xiangya Hospital, Central South University, Changsha, Hunan, P. R. China

## Abstract

Lipid raft proteins have been confirmed to be important in cell signal transduction. Some reports have shown that the aberrant expression of lipid raft proteins is associated with malignant phenotypes in some cancers. However, the role of the lipid raft protein flotillin-2 (Flot-2) in nasopharyngeal carcinoma (NPC) remains to be comprehensively characterized. Here, overexpression of Flot-2 in NPC tissues and cell lines was detected by immunostaining, and Flot-2 expression was found to be positively associated with NPC metastasis. Furthermore, inhibiting Flot-2 expression impaired the malignancy of the highly metastatic NPC cell line 5-8F by constraining its growth and proliferation, mobility and migration, and decreasing the capacity of 5-8F cells to metastasize in nude mice. In contrast, forced overexpression of Flot-2 increased the malignancy of 6-10B, a non-metastatic NPC cell line that weakly expresses Flot-2. Moreover, in 5-8F-shFlot-2 cells, which have inhibited Flot-2 expression, the NF-κB and PI3K/Akt3 pathways were inactivated. Subsequently, MMPs expression were decreased, and Foxo1 activity was increased. In addition, enhanced NF-κB and PI3K/Akt3 activities were observed in Flot-2 overexpressing 6-10B cells. Thus, Flot-2 exerts a pro-neoplastic role in NPC and is involved in tumor progression and metastasis. Moreover, Flot-2 exerts its role through NF-κB and PI3K/Akt3 signaling.

Metastasis is one of the primary obstacles to effective therapy for tumors, and over 90% of deaths of patients with solid tumors result from metastasis^1^,^2^. Metastasis is the result of a complex cascade of events, including transformation, angiogenesis, mobility, and invasion. Tumor cells must manipulate the functions of numerous biological processes to achieve successful metastasis. Of these processes, cell membrane modification plays a vital role in initiating cell migration. Lipid rafts are specialized heterogeneous microdomains found in the plasma membrane and have been demonstrated to exert their influence in many physiological and pathological processes such as cancer metastasis^3^,^4^,^5^.

Flotillins are key components of lipid rafts and belong to the stomatin/prohibitin(PHB)/flotillin/HflK/C(SPFH) domain-containing protein family. There are two flotillin members of this family: flotillin-1 (Flot-1) and flotillin-2 (Flot-2)^5^. These proteins can stabilize each other by forming a hetero-oligomer^6^. Flotillins may play important roles in cancer development as positive regulators. A high level of expression of Flot-1 or Flot-2 can enhance tumor growth and tumor cell migration. Flot-1 and Flot-2 are considered to be candidate markers for lymph node metastasis and for predicting poor prognosis and may be useful therapeutic targets for some types of cancers^7^,^8^,^9^,^10^,^11^,^12^,^13^. Furthermore, reduced Flot-2 expression was shown to result in a reduction in lung metastases of breast cancer in a mouse breast cancer model^12^.

Nasopharyngeal carcinoma (NPC) is a type of malignant head and neck tumor. NPC is mainly prevalent in southeast Asia and coastal regions of China^14^. Radiation therapy may be used as a treatment alone or in combination with chemotherapy and surgery^15^. Distant metastasis is very common and is the main cause of death of NPC patients^15^. Our previous study revealed that NPC tumor cells with high Flot-2 expression have a high metastatic potential, indicating that Flot-2 may be involved in NPC metastasis^16^. A recent study also revealed the correlation between Flot-2 expression and lymph node metastasis in NPC patients^17^. However, the roles of Flot-2 in NPC are largely unknown.

In this study, we investigated Flot-2 expression in NPC cell lines and NPC tumor tissues and further explored the roles of Flot-2 in the development of NPC and the underlying mechanisms.

## Results

### Flot-2 expression was positively associated with the progression of NPC

**Figure 1:**
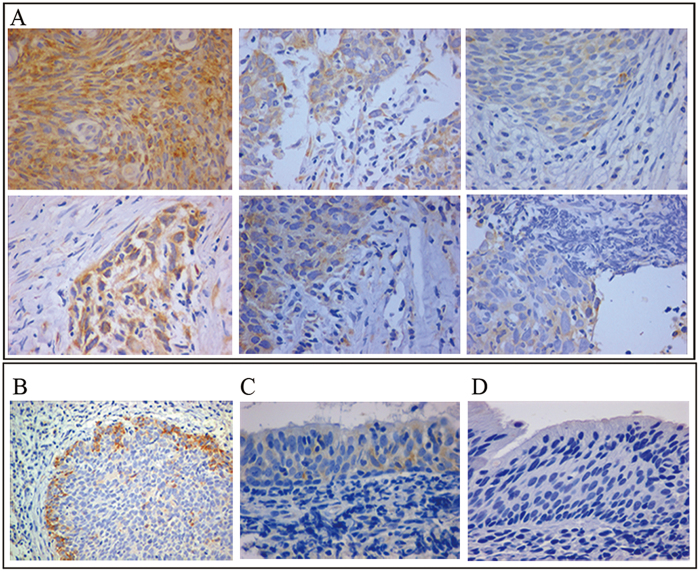
Immunostaining of Flot-2 in clinical NPC and NP tissues. **A**, Flot-2 showed a diffuse staining pattern with different intensities in metastatic (upper panel) and non-metastatic (lower panel) NPC tissues. **B**, Flot-2 showed a focal expression pattern at the periphery of NPC nests with no or weak expression in the central areas. **C**, NP tissues with faint Flot-2 expression. **D**, NP tissues with negative Flot-2 expression. The histological manifestations shown in Fig. 1 are representative cases.

Flot-2 staining was mainly located at the membrane and in the cytoplasm of epithelial cells. Flot-2 expression was generally heterogeneous in NPC tumor tissues, with two different patterns: diffuse expression in most living tumor cells (Fig. 1A) and focal expression at the proliferating periphery of tumor nests (Fig. 1B). Positive Flot-2 expression was detected in all NPC tissues. In contrast, Flot-2 expression was not detectable (30/38) (Fig. 1D) or was detected at low levels (8/38) in the basal cells of nasopharynx (NP) tissues (Fig. 1C). Both the positive expression rate and the intensity of Flot-2 expression in metastatic NPC tissues were also significantly higher than those in non-metastatic NPC tissues (Table 1). These findings suggest that overexpression of Flot-2 is related to the occurrence of NPC and promotes NPC invasion and metastasis.

### The expression pattern of Flot-2 in NPC cell lines

**Figure 2:**
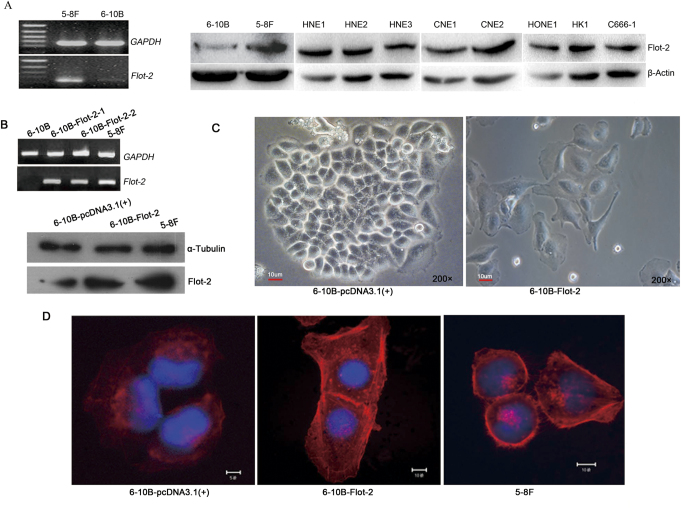
The effect of Flot-2 overexpression on the morphology of 6-10B cells. **A**, The Flot-2 expression level in 5-8F, 6-10B and other NPC cells was detected by semi-quantitative RT-PCR and Western blotting. The expression of Flot-2 in 6-10B was weaker than that in other NPC cells. **B**, Semi-quantitative RT-PCR and Western blotting were used to detect Flot-2 expression in 6-10B-Flot-2 cells. The 6-10B-Flot-2 cells achieved a comparable Flot-2 expression level to that in 5-8F cells. **C**, The morphology of 6-10B and 6-10B-Flot-2 cells observed by inverted microscopy (200×). 6-10B-Flot-2 cells had a mesenchymal-like morphology with lamellipodia. **D**, Cytoskeleton of 6-10B, 6-10B-Flot-2 cells and 5-8F cells were recorded under confocal laser-scanning microscope. 6-10B-Flot-2 cells exhibited a similar microfilament distribution pattern to 5-8F cells, consisting of a high-density distribution of microfilaments on the cell surface, which precedes the formation of conspicuous lamellipodia and membrane ruffles.

RT-PCR and Western blotting revealed ubiquitous expression of Flot-2 in all NPC cell lines included in this study. Flot-2 expression was significantly higher in 5-8F cells than in 6-10B cells (Fig. 2A). Both 5-8F and 6-10B cells were isolated from SUNE-1 cells. They have a similar genetic background but differ in their metastatic ability—5-8F cells are highly metastatic, whereas 6-10B cells are non-metastatic^18^. The expression of Flot-2 in non-metastatic 6-10B cells was also clearly lower than that in other NPC cells with metastatic potential (Fig. 2A). This result may imply that Flot-2 is associated with the metastatic feature of NPC tumors.

### Upregulating Flot-2 expression promotes malignancy of 6-10B cells both *in vitro* and *in vivo*

A 6-10B cell line stably expressing Flot-2 (6-10B-Flot-2) was successfully established by transfecting 6-10B cells with a pcFlot-2 expression vector (Fig. 2B). 6-10B-Flot-2 cells exhibited a Flot-2 expression level that was comparable with that of 5-8F cells. 6-10B cells transfected with pcDNA3.1(+) empty vector (6-10B-pcDNA3.1(+)), were used as a control. The influences of ectopic Flot-2 expression on the biological characteristics of 6-10B cells were analyzed both *in vitro* and *in vivo*.

**Figure 3:**
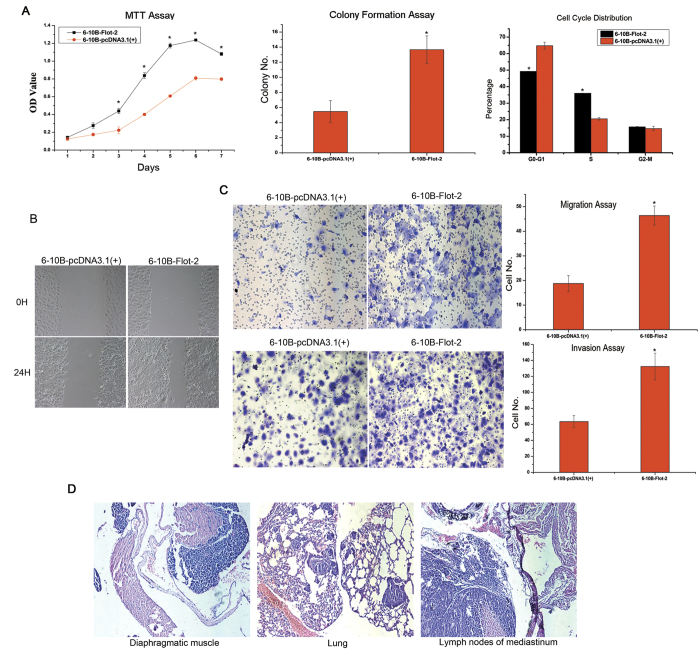
The effect of Flot-2 overexpression on the biological characteristics of 6-10B-Flot-2 cells. **A**, MTT assay, colony formation assay and flow cytometric analysis were carried out to analyze the influence of ectopic Flot-2 expression on the growth, proliferation, and cell cycle stage of 6-10B cells. Enhanced proliferation (significant differences were observed since Day3), colony formation (colony number: 6-10B-pcDNA3.1(+): 5.48 ± 1.44, 6-10B-Flot-2: 13.67 ± 2.45) and cell cycle progression(S stage ratio(%): 6-10B-pcDNA3.1(+): 36.01 ± 0.08, 6-10B-Flot-2: 20.54 ± 0.79) were observed for 6-10B-Flot-2 cells. **B**, Effects of Flot-2 on cell motility of 6-10B cells measured by an *in vitro* scratch wound healing assay. **C**, The influences of Flot-2 on the motility and *in vitro* invasiveness of 6-10B cells measured by a migration assay (cell number: 6-10B-pcDNA3.1 (+):16.7 ± 3.34, 6-10B-Flot-2: 46.58 ± 4.35) and an *in vitro* Matrigel invasion assay (cell number: 6-10B-pcDNA3.1 (+): 63.75 ± 8.13, 6-10B-Flot-2: 132.21 ± 17.63). 6-10B-Flot-2 cells displayed significantly stronger migratory and invasive capacities than that of 6-10B-pcDNA3.1 (+) cells *in vitro*. **D**, *In vivo* metastasis assay of 6-10B-Flot-2 cells. After intraperitoneal inoculation, 6-10B-Flot-2 cells invaded into diaphragmatic muscle, metastasized to lung and formed multiple metastases, and metastasized to the mediastinal lymph nodes. Thus, the invasive and metastatic capacities of 6-10B-Flot-2 were also confirmed *in vivo*. All data were representative of three independent experiments. The data were analyzed by a two-tailed student *t*-test.* indicates *P* < 0.05.

Enhanced Flot-2 expression caused dramatic changes in the morphology of 6-10B cells, including the expansion of cells, a decrease in the nucleo-cytoplasmic ratio, and the formation of lamellipodia, resulting in morphological properties similar to mesenchymal cells but distinct from classic epithelial cells (Fig. 2C). Cytoskeleton staining revealed that 6-10B-Flot-2 cells exhibited a similar pattern of microfilament distribution to 5-8F cells in that microfilaments were densely distributed on the cell surface, which indicates cellular preparation for the formation of conspicuous lamellipodia and membrane ruffles (Fig. 2D). At the same time, ectopic Flot-2 expression resulted in more aggressive proliferation of 6-10B cells, reflected by the formation of larger and more numerous colonies and faster growth, probably by increasing the percentage of cells in the S phase (Fig. 3A).

In addition, the migratory and invasive abilities of 6-10B-Flot-2 cells were also significantly increased, as demonstrated by increased wound closure in a scratch wound healing assay (Fig. 3B), an increased migration rate in a transwell migration assay and an enhanced invasion rate in a Matrigel invasion assay (Fig. 3C). Moreover, intraperitoneal injection of 6-10B-Flot-2 cells not only induced primary tumor nodules on the surface of abdominal organs (the diaphragm, pancreas, porta, spleen, and mesentery) in nude mice but also caused the development of distant metastases, including metastases in the lungs and mediastinal lymph nodes (Fig. 3D). However, metastasis was not observed in 6-10B-pcDNA3.1(+) injected mice (only primary tumors were induced).

### Knockdown of Flot-2 impaired the metastatic ability of 5-8F cells

**Figure 4:**
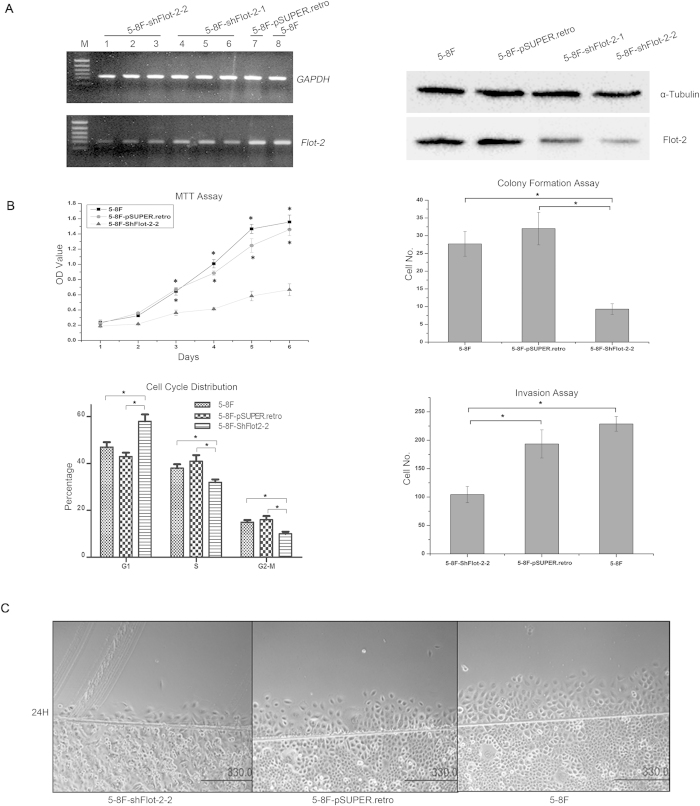
The effects of Flot-2 knockdown on biological characteristics of 5-8F cells. **A**, Stable Flot-2 knockdown was successfully established in 5-8F cells, reflected by RT-PCR and Western blotting. Analysis showed the expression level of Flot-2 was significantly downregulated (more than 75%) in 5-8F-shFlot-2-2 cells. **B**, Flot-2 knockdown resulted in slower growth(significant differences were observed since Day3) and proliferation(colony number: 5-8F-shFlot-2: 9.3 ± 1.52, 5-8F-pSUPER.retro: 32 ± 4.58, 5-8F: 27.7 ± 3.51), cell cycle arrest (S stage ratio(%): 5-8F-shFlot-2: 30.49 ± 2.23, 5-8F-pSUPER.retro: 42.58 ± 3.56, 5-8F: 40.43 ± 3.67) and impaired invasiveness of 5-8F cells (cell number: 5-8F-shFlot-2: 106.25 ± 13.28, 5-8F-pSUPER.retro: 185.32 ± 30.54; 5-8F: 227.41 ± 17.46), measured by MTT assay, soft agar colony formation assay, FACS analysis and *in vitro* Matrigel invasion assay. **C**, Scratch wound healing assay demonstrated that Flot-2 knockdown could decrease the motility of 5-8F cells. All data were representative of three independent experiments. The data were analyzed by a two-tailed student *t*-test. * indicates *P* < 0.05.

We further investigated whether downregulating Flot-2 expression in 5-8F cells could exert a negative effect on their metastatic ability. Two cell lines, 5-8F-shFlot-2-1 and 5-8F-shFlot-2-2, were established by introducing two short hairpin RNA expression cassettes (shFlot-2-1 and shFlot-2-2) into 5-8F cells to silence Flot-2 expression. Compared with 5-8F-shFlot-2-1 cells, 5-8F-shFlot-2-2 cells had a lower level of Flot-2 expression, detected by RT-PCR and Western blotting (Fig. 4A). Thus, we used 5-8F-shFlot-2-2 cells in the following studies and designated them as 5-8F-shFlot-2 cells. The 5-8F-pSUPER.retro blank vector transfected 5-8F cells were established as a control cell line.

The MTT assay and the colony formation assay showed that the proliferation and colony formation abilities were markedly restrained in 5-8F-shFlot-2 cells compared with 5-8F cells and 5-8F-pSUPER.retro cells (Fig. 4B). FACS analysis showed that reduced Flot-2 expression delayed the G1 to S progression (Fig. 4B), demonstrating that Flot-2 knockdown may inhibit 5-8F cell proliferation by inducing cell cycle arrest.

**Figure 5:**
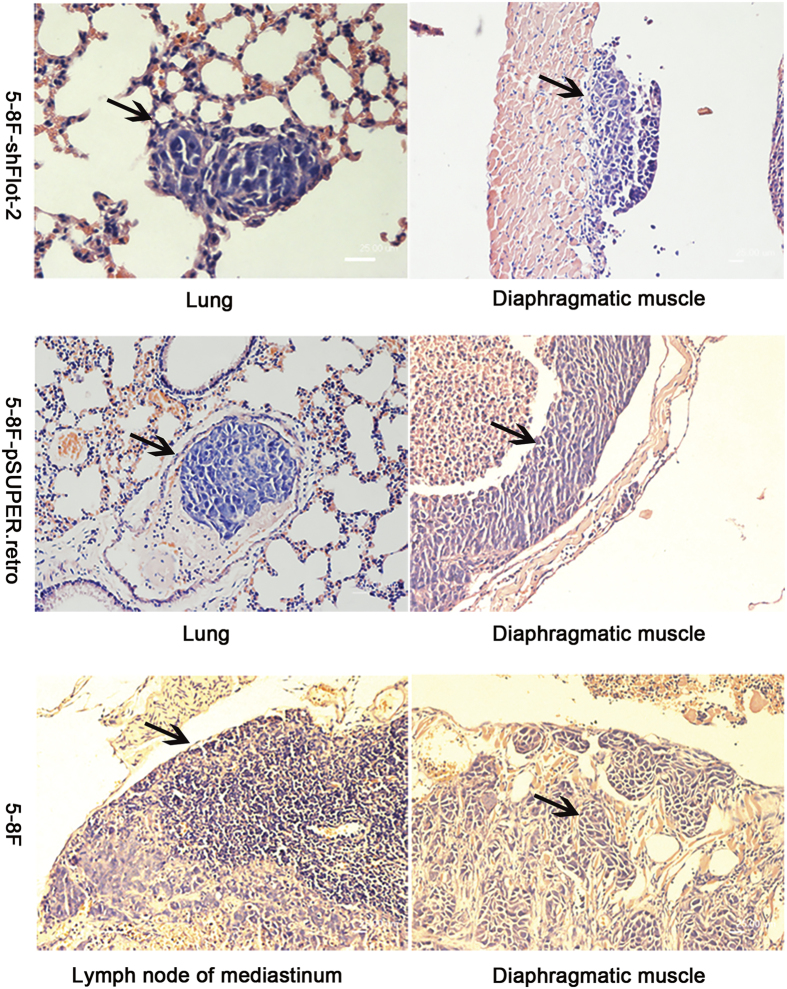
*In vivo* metastasis assay of 5-8F-shFlot-2 cells. Only one mouse (1/5) developed a metastasis of 5-8F-shFlot-2 cells, which metastasized to lung and formed only micro-metastases (representative photo is shown in the upper left corner). Four mice (4/5) inoculated with 5-8F-pSUPER.retro cells developed metastases in the mediastinal lymph nodes and lungs. In the lung tissues, tumor embolus could be easily found (representative photo is shown in the middle left picture). All mice (5/5) inoculated with 5-8F cells developed metastases in the mediastinal lymph nodes and lungs. A representative photo showing the metastasis to the mediastinal lymph node is presented here (the lower left corner). After intraperitoneal inoculation, 5-8F-shFlot-2 cells migrated to the diaphragmatic muscle without obvious invasion (the upper right corner), whereas 5-8F-pSUPER.retro cells (the middle right photo) and 5-8F cells (the lower right corner) invaded deeply into the diaphragmatic muscle. The *in vivo* invasion and metastatic ability of 5-8F cells was inhibited by Flot-2 knockdown.

Furthermore, the *in vitro* scratch wound healing assay and the Matrigel invasion assay revealed that inhibition of Flot-2 expression significantly decreased the mobility and invasive capacity of 5-8F cells (Fig. 4C,B). *In vivo* analysis (Fig. 5) showed that 5-8F-shFlot-2 cells formed less distant metastases, with only one mouse developing a distant metastasis (1/5). In contrast, a high rate of metastasis was observed in mice inoculated with 5-8F cells (5/5) and 5-8F-pSUPER.retro cells (4/5). From these results, it can be concluded that downregulated Flot-2 expression impairs the metastastic ability of 5-8F cells.

### Microarray analysis in Flot-2 silenced NPC cells

To explore the molecular mechanisms of Flot-2 in NPC, cDNA microarray analysis was conducted in 5-8F-pSUPER.retro cells and 5-8F-shFlot-2 cells. The expression of 481 genes was upregulated and the expression of 232 genes was downregulated in 5-8F-shFlot-2 cells compared with 5-8F-pSUPER.retro cells (Supplementary Table 1). These genes are predicted to be involved in many biological processes such as cell adhesion, signal transduction, and immune response. The microarray analyses were further validated by confirming the expression levels of randomly selected genes (both upregulated and downregulated) using qPCR (Supplementary Figure 1). The microarray data can be achieved from GEO Datesets database with accession number GSE67456.

### Flot-2 knockdown led to downregulated expression of MMPs, most likely by inhibiting the activity of the NF-κB signaling pathway

**Figure 6:**
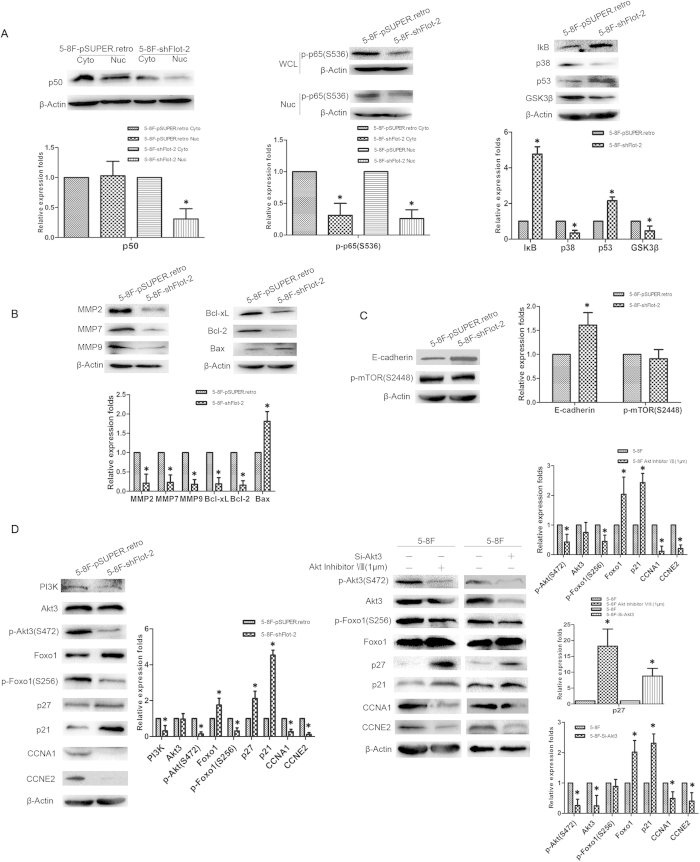
Western blotting was used to compare the activities of NF-κB and PI3K/Akt3 signaling between 5-8F-pSUPER.retro and 5-8F-shFlot-2 cells. **A**, Direct or indirect evidence for inactivation of NF-κB in 5-8F-shFlot-2 cells. Cyto, cytoplasm; Nuc, nucleus; WCL, whole cell lysate. The reduced expression levels of nuclear p50 and phosphorylated p65 in 5-8F-shFlot-2 cells were direct evidence for the inactivation of NF-κB. Enhanced IκB and p53 expression combined with lower p38 and GSK3β expression were indirect evidence. **B**, Detecting the expression of downstream effectors of NF-κB including MMP2, 7, 9, Bcl-xL, Bcl-2, and Bax. The expression of MMPs, Bcl-2 and Bcl-xL was reduced and the expression of Bax was enhanced in 5-8F-shFlot-2 cells, compared with 5-8F-pSUPER.retro cells. **C**, Detecting the expression of E-cadherin and p-mTOR in 5-8F-shFlot-2 cells. The expression of E-cadherin was upregulated in 5-8F-shFlot-2 cells, and there was no difference in expression of p-mTOR between 5-8F-shFlot-2 cells and 5-8F-pSUPER.retro cells. **D**, Reduced activity of the PI3K/Akt3/Foxo1 signaling axis in 5-8F-shFlot-2 cells was confirmed by RNA interference and Akt Inhibitor VIII treatment. Flot-2 knockdown impaired the PI3K/Akt3/Foxo1 axis in 5-8F cells, as demonstrated by inhibited expression of PI3K, p-Akt3, p-Foxo1, CCNA1 and CCNE2 and enhanced expression of p27, p21 and Foxo1. Similar outcomes were obtained in 5-8F cells treated by Akt3 Inhibitor VIII and Akt3 knockdown in 5-8F cells. All data were representative of three independent experiments. The gels have been run under the same experimental conditions and the original pictures for clipped ones like E-cadherin, MMPs, p-Akt3, NF-κB factors and p-Foxo1 were showed in suppleplementry figs 2. The western blot bands were quantified and analyzed by a two-tailed student *t*-test. * indicates *P* < 0.05.

Western blot analysis showed that the expression of MMP2, MMP7 and MMP9 were clearly downregulated in 5-8F-shFlot-2 cells (Fig. 6B). The activation of NF-κB signaling has been revealed to regulate the expression of MMPs in some tumors^19^,^20^. Therefore, we further detected the activation status of key regulators in NF-κB signaling. Decreased activity of NF-κB was observed in 5-8F-shFlot-2 cells and was directly illustrated by the translocation of p50 from the nucleus (active) to the cytoplasm (inactive), a lower level of phospho-p65 in the nucleus, and the upregulation of IκB (Fig. 6A). Accordingly, alterations in the expression of upstream regulators of NF-κB, such as p53^21^, p38^22^, and GSK3β^23^ also confirmed the inactivation of NF-κB (Fig. 6A). In addition, the altered expression patterns of Bcl-xL, Bcl-2, and Bax (Fig. 6B), which are proteins involved in cell survival and targeted by NF-κB, are an additional piece of evidence indicating downregulated activity of NF-κB signaling. Collectively, it appears that the downregulation of Flot-2 weakened the expression of MMPs by inactivating NF-κB signaling, which subsequently decreased the migratory capacity of NPC cells.

### Akt3 and Foxo1 level in 5-8F-shFlot-2 cells

Western blot analysis confirmed the downregulated expression of CCNA1 and CCNE2 and upregulated expression of CDKN1A (also named p21) in 5-8F-shFlot-2 cells observed in the microarray analysis (Fig. 6D). The FACS analysis above had revealed that the 5-8F-shFlot-2 cells were arrested in G1/S phase, suggesting an association between cell cycle regulation and Flot-2 expression. In light of this result, the activity of Foxo1, a key negative regulator of the cell cycle was detected in both 5-8F-pSUPER.retro cells and 5-8F-shFlot-2 cells. Reduced expression of phospho-Foxo1 (inactive form) was conspicuous in 5-8F-shFlot-2, which then increased the expression of several important negative cell cycle regulators such as p21 and p27 (Fig. 6D). The PI3K/Akt signaling axis is the main upstream pathway that regulates the activity of Foxo1^24^. Indeed, the expression of PI3K was inhibited in 5-8F-shFlot-2 cells (Fig. 6D). However, the levels of neither Akt1 nor Akt2, the two PI3K effectors in the majority of circumstances, were altered in 5-8F-shFlot-2 cells. In contrast, phospho-Akt3 expression was significantly decreased (Fig. 6D). An Akt-specific inhibitor, Akt Inhibitor VIII, and siRNA-mediated knockdown of Akt3, were applied to confirm the effects of Akt3 in 5-8F cells. The activity of Foxo1 was augmented by the inhibition of Akt activity by Akt Inhibitor VIII (1 μM) or knockdown of Akt3 expression (Fig. 6D). We also detected the activity of mTOR and found that the p-mTOR level was similar between 5-8F-pSUPER.retro and 5-8F-shFlot-2 cells (Fig. 6C), thereby excluding the influence of the PI3K/Akt/mTOR axis. Thus, these findings suggest that Flot-2 may activate Akt3, which then inhibits Foxo1 and promotes the progression of the cell cycle. In addition, upregulated expression of E-cadherin has been demonstrated to repress metastasis in oral squamous cell carcinoma cells with inhibited Akt activity^25^. A similar outcome was observed in 5-8F-shFlot-2 cells (Fig. 6C), suggesting that Flot-2 depletion could impair the metastatic ability of 5-8F cells by enhancing E-cadherin expression.

### Flot-2 overexpression enhanced the activity of PI3K/Akt3 and NF-κB in 6-10B cells

**Figure 7:**
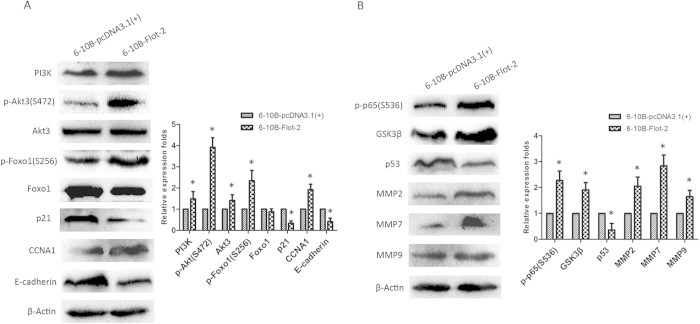
The activities of NF-κB and PI3K/Akt3 pathways were reinforced in 6-10B-Flot-2 cells. **A**, Enhanced activity of the PI3K/**A**kt3 axis was confirmed in 6-10B-Flot-2 cells by detecting upregulated expression of PI3K, p-Akt3 and Akt3. Additional evidence includes the altered expression of downstream effectors, such as upregulated p-Foxo1 and CCNA1 and downregulated Foxo1, p21 and E-cadherin. **B**, The NF-κB pathway was also activated in 6-10B-Flot-2 cells, reflected by upregulated p-p65, GSK3β and MMPs and downregulated p53. All data were representative of three independent experiments. The gels have been run under the same experimental conditions and the original pictures for clipped ones like E-cadherin, MMPs, p-Akt3, NF-κB factors and p-Foxo1 were showed in suppleplementry figs 2. The western blot bands were quantified and analyzed by a two-tailed student *t*-test. * indicates *P* < 0.05.

To verify that Flot-2 knockdown could inhibit the activity of PI3K/Akt3 and NF-κB, from a different perspective, we analyzed the influences of Flot-2 overexpression on 6-10B cells. Here, the expression of phospho-Akt3, PI3K, phospho-p65 and phospho-Foxo1 was increased in 6-10B-Flot-2 cells (Fig. 7A,B). Accordingly, the expression of CCNA1, MMP2, MMP7, MMP9, GSK3β was also increased (Fig. 7A,B). However, the negative regulators of tumor growth, such as Foxo1, p21, E-cadherin, and p53, were suppressed (Fig. 7A,B). Therefore, enhanced malignancy of 6-10B-Flot-2 cells may be due to the increased activity of PI3K/Akt3 and NF-κB resulting from the overexpression of Flot-2.

### Flot-2 can interact with Flot-1 and shows a positively related expression pattern in NPC cells

**Figure 8:**
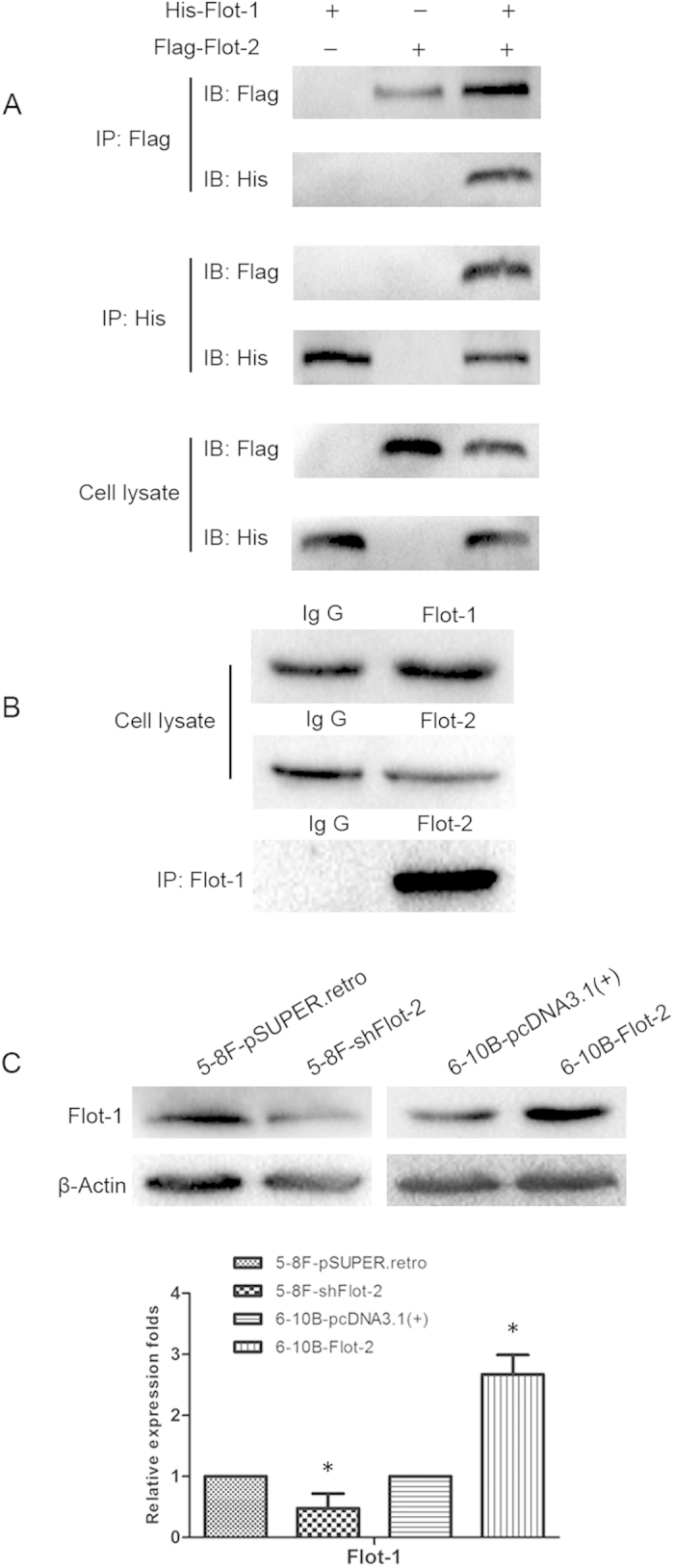
Analysis of the interaction between Flot-2 and Flot-1 both in 293T and 5-8F cells, and expression patterns of Flot-1 in 5-8F-shFlot-2 and 6-10B-Flot-2 cells. **A**, positive bands of anti-Flag (Flot-2) or anti-His (Flot-1) were detected in complexes immunoprecipitated by anti-His or anti-Flag, respectively, in 293T cells. **B**, A positive band of Flot-2 was detected in a complex immunoprecipitated by anti-Flot-1 in 5-8F cells. **C**, Flot-1 expression was positively associated with that of Flot-2, as demonstrated by downregulated Flot-1 in 5-8F-shFlot-2 cells and upregulated Flot-1 in 6-10B-Flot-2 cells. All data were representative of three independent experiments. The western blot bands were quantified and analyzed by a two-tailed student *t*-test. * indicates *P* < 0.05.

The positive relationship in the expression patterns of flotillins, (*i.e.*, decreased or increased expression of one leads to the same expression pattern of the other), has been observed in both cells and knockout mouse models^6^,^26^,^27^. Here, we first confirmed the interaction of Flot-1 and Flot-2 in 293T cells (Fig. 8A) and 5-8F cells (Fig. 8B) by co-immunoprecipitation (co-IP). Then, we detected whether flotillin-2 knockdown or overexpression affects the expression of its counterpart, flotillin-1. We found that the expression of Flot-2 and Flot-1 was positively correlated in NPC cells, as demonstrated by increased or decreased Flot-1 expression in 6-10B-Flot-2 or 5-8F-shFlot-2 cells, respectively (Fig. 8C). Thus, the positive correlation between Flot-2 and Flot-1 suggested that Flot-2 may be involved in the stability of Flot-1 and Flot-1 may play a certain role in the outcome of Flot-2 alterations, though these require further investigation.

## Discussion

In this study, overexpression of Flot-2 was observed both in NPC biopsies and cell lines. Flot-2 knockdown impaired the malignancy of 5-8F cells, as demonstrated by their reduced capacity to form colonies, migrate and invade *in vitro*, as well as to metastasize in nude mice. Silencing Flot-2 expression in 5-8F cells inhibited NF-κB and PI3K/Akt3 signaling and subsequently decreased MMPs expression, increased E-cadherin expression, enhanced Foxo1 activity and induced cell cycle arrest. To exclude the possibility of clonal variance, we also detected the influence of Flot-2 knockdown on 5-8F-shFlot-2-1 cells, and we observed similar outcomes (data not shown). These findings provide strong evidence that Flot-2 plays a significant role in promoting NPC progression through the regulation of NF-κB and PI3K/Akt3 signaling.

The roles of flotillins in cancer progression have been studied in various cancers. Knockdown of Flot-1 or Flot-2 by RNAi inhibits the proliferation, invasion, migration and metastasis of cancerous cells. Both Flot-1 and Flot-2 are promising markers for the diagnosis and prediction of outcome for some cancers^10^,^11^,^17^. Here, we observed the overexpression of Flot-2 in NPC tissues and cell lines, and the expression pattern of Flot-2 was positively correlated with NPC metastasis, a finding that is supported by recent work that indicates that Flot-2 can serve as a novel biomarker for lymph node metastasis in NPC. In accordance with the findings in other cancers^13^,^28^, knockdown of Flot-2 inhibited the proliferation, mobility and invasion abilities of 5-8F cells. Overexpression of Flot-2 in 6-10B cells resulted in mesenchymal-like morphology and enhanced invasive and metastatic ability. With these results, we have demonstrated that Flot-2 can also promote progression of NPC, similar to other tumors.

Breaking through the extracellular matrix and basement membrane is an essential step for the metastasis of cancerous cells^2^,^29^. Increased expression of MMPs plays a vital role in disrupting the extracellular barriers, which then facilitates the migration of cancerous cells^30^. The positive correlation between Flot-2 and metastasis in breast cancer^8^, melanoma^11^, and gastric cancer^31^ as well as NPC^17^, were demonstrated by clinical pathological analysis. The pro-metastatic role of Flot-2 was demonstrated in a mouse breast cancer model^12^. Activation of NF-κB signaling, observed in most cancers, has been shown to contribute to cancer occurrence and progression by regulating multiple processes, including cell survival and proliferation, EMT (epithelial to mesenchymal transition), inflammation, and angiogenesis, as well as metastasis^19^,^20^. Activated NF-κB has been verified to upregulate expression of MMPs in a variety of metastatic cancers^32^,^33^. The suppression of NF-κB activity and inhibition of metastasis resulting from downregulated Flot-1 have been confirmed in esophageal and oral squamous cell carcinomas^7^,^34^. However, the relationship between Flot-2 and NF-κB has been rarely analyzed. Here, the downregulated and upregulated activities of NF-κB/MMPs in 5-8F-shFlot-2 and 6-10B-Flot-2 cells, respectively, demonstrated that the pro-metastatic role of Flot-2 in NPC might result from its ability to promote MMPs expression by activating NF-κB.

The PI3K/Akt signaling pathway plays an important role in fundamental intracellular signaling transduction systems. It regulates various cellular and physiological activities such as cell growth, proliferation, apoptosis, angiogenesis and metabolism by phosphorylation of various downstream effectors such as Foxo1 and mTOR^35^,^36^. However, aberrant activation of PI3K/Akt signaling contributes to tumorigenesis and metastasis^37^,^38^. Although the three isoforms of Akt, Akt1, Akt2 and Akt3, share considerable homology, distinct regulatory functions of Akts have been identified in different types of cancers, which are summarized in detail in Romano G’s review^39^. Inhibition of Akt1/Foxo3a/p21/p27 was observed in breast cancer cells with Flot-1 knockdown, which was associated with an arrest of proliferation and tumorigenicity^9^. Surprisingly, only Akt3, whose role in NPC has not yet been reported, acted as an effector of PI3K and was involved in the regulation of cell cycle control at G1/S checkpoint in NPC cells. Flot-2 knockdown did not impair the activity of mTOR, which excluded the role of the PI3K/Akt/mTOR signaling axis, an important signaling pathway in the progression of tumors^36^,^40^. The hyperactivity of Akt3 has been observed in some cancers, including breast cancer^41^, melanoma^42^, ovarian cancer^43^, and hepatocellular carcinoma^44^, and it is involved in the progression of the above mentioned tumors by regulating different downstream targets. This study confirmed the direct association between lipid rafts and PI3K/Akt signaling reported in mantle cell lymphoma^45^ and breast cancer^9^ and indicated a possible Flot-2/PI3K/Akt3 signaling pathway.

Flotillins tend to form hetero or homo-oligomers to stabilize each other^6^,^46^. Depletion of either Flot-1 or Flot-2 expression can concomitantly decrease the expression of the other in both Flot-1 or Flot-2 knockout mice and cultured cells. It seems that Flot-1 is more dependent on Flot-2 because Flot-1 depletion typically causes little to no depletion of Flot-2, whereas Flot-2 knockdown not only reduces the expression of Flot-1 but also completely depletes the flotillin-specific membrane microdomains, a key platform for signal transduction^6^,^12^. Thus, Flot-2 knockout inhibits lung metastasis in a breast cancer mouse model^12^. Here, a similar mechanism may be adopted in NPC, as decreased Flot-1 expression was observed in 5-8F-shFlot-2 cells and increased Flot-1 expression was observed in 6-10B-Flot-2 cells. Further work is urgently needed to elucidate the underlying relationship of flotillins in NPC.

## Conclusion

In conclusion, this study demonstrated that Flot-2 exerts a cancerous role in NPC and is involved in tumor progression and metastasis. Flot-2 exerts its role in NPC tumors through NF-κB and PI3K/Akt3 signaling. Therefore, we can speculate that upregulation of Flot-2 activates NF-κB, which subsequently increases the expression of MMPs, degrades the extracellular matrix, and finally promotes the metastasis of NPC cells. In addition, upregulation of Flot-2 activates PI3K/Akt3 and inhibits Foxo1 activity, leading to an acceleration of the cell cycle through downstream effectors of Foxo1 and subsequently to proliferation of NPC cells.

## Materials and Methods

### Cell lines and tissues

NPC cell lines (5-8F, 6-10B, CNE1, CNE2, HNE1, HNE2, HNE3, HK1, C666-1, HONE1) were maintained by our laboratory. Cells were grown in RPMI 1640 (Invitrogen, Carlsbad, CA, USA) supplemented with 10% FBS in a humidified atmosphere with 5% CO_2_ at 37 °C. Thirty-eight NP tissues and 132 primary NPC tissues, including 45 non-metastatic and 87 metastatic NPC tissues, were used for analysis of Flot-2 protein expression by immunohistochemistry (IHC). All samples were obtained from patients before treatment at Hunan Cancer Hospital (Changsha, Hunan, China) with their informed consent. The study was carried out after approval by the Ethics Committee of Central South University. The methods were carried out in accordance with the approved guidelines.

### Immunohistochemistry (IHC)

Tissue slides were immunoreacted with anti-Flot-2 mouse monoclonal antibody (1:50, Santa Cruz Biotechnology, USA) and detected by IHC using the SAB (streptavidin-biotin) system (DAKO, Carpinteria, CA). Sections were independently evaluated and scored by two pathologists who were blinded to the clinical data. Evaluation of staining was assessed using the Intensity Reactivity Score (IRS), according to the literature^47^.

### Forced expression of Flot-2 in 6-10B cells and knockdown of Flot-2 or Akt3 in 5-8F cells

The open reading frame (ORF) sequence of Flot-2 was amplified from 5-8F cell cDNA using the forward primer 5'-AAACGGGTGCTGGAGGGAGGGC-3' and the reverse primer 5'-CTGGGGGTGGCGGGATAGGCTG-3' and subcloned into the pcDNA3.1(+) vector. Two RNAi sequences targeting Flot-2, 5'-ATGACAAAGTGGACTATCT-3' and 5'-AAGGCAGAAGCCTACCAGAAA-3' (named shFlot-2-1 and shFlot-2-2, respectively), were cloned into the pSUPER.retro vector system as previously described^48^ and confirmed by DNA sequencing. 6-10B cells or 5-8F cells were transfected with 2 μg of corresponding vectors by Lipofectamine 2000^TM^ reagent (Invitrogen) according to the manufacturer’s protocols. After two weeks of selection with G418 (6-10B) or puromycin (5-8F), drug-resistant clones were obtained and the expression of Flot-2 was confirmed by RT-PCR and Western blotting. Then, the acquired cells were named 6-10B-Flot-2, 5-8F-shFlot-2-1 and 5-8F-shFlot-2-2. The control cells were obtained in a similar way. Knockdown of Akt3 was performed according to previously published protocol^49^.

### Semiquantitative or quantitative reverse transcription-PCR (RT-PCR or qPCR)

RT-PCR and qPCR reactions were performed as described previously. Glyceraldehyde-3-phosphate dehydrogenase (GAPDH) was used as an internal control. Flot-2 was amplified using the forward primer 5'-GGCTTGTGAGCAGTTTCTGG-3' and the reverse primer 5'-TCGAAGGCTCGCTTAGAGTC-3'. GAPDH was amplified using the forward primer 5'-ACCACAGTCCATGCCATCAC-3’ and the reverse primer 5'-TCCACCACCCTGTTGCTGT-3'. The primers for qPCR were provided in Supplementary Table 2.

### Western blot analysis

Western blotting was carried out as previously described with a minor modification. The antibodies used in the study are as follows: rabbit polyclonal anti-MMP2, anti-MMP7, anti-MMP9, anti-Bcl-xl, anti-Bcl-2, anti-Bax, anti-E-cadherin, anti-PI3K, anti-p53, anti-GSK3β, anti-Flot-1, anti-Akt3 (Proteintech, Wuhan, China), anti-phospho-Akt3 (S472) (Abgent, Suzhou, China), anti-phospho-p65 (S536), p50, anti-IκB, anti-Foxo1, anti-phospho-Foxo1 (S256), anti-p38, anti-p27, anti-p21, anti-CCNA1, anti-CCNE2 (Sangon Antibody R&D Center, Shanghai, China), anti-phospho-mTOR (S2448) (ImmunoWay, Newark, DE, USA), mouse monoclonal anti-Flot-2, anti-α-tubulin (Santa Cruz Biotechnology, Santa Cruz, CA, USA), and anti-β-actin (Sigma, USA). Akt Inhibitor VIII, a specific Akt inhibitor, was purchased from Merck Millipore (Merck KGaA, Darmstadt, Germany). Quantification of signal intensity (IOD, integral optical density) was performed with Gel-Pro Analyzer software(Version 4.0). Expression change was indicated by IOD ratio of targeted protein before and after treatments. And the intensity was normalized by β-Actin signal. All detections were repeated for three independent times.

### Co-immunoprecipitation (Co-IP)

pEF1/myc-His-Flot-1 and pFLAG-CMV-Flot-2 expression vectors were constructed by cloning the Flot-1 ORF and Flot-2 ORF into pEF1/myc-His vector (Invitrogen, USA) and pFLAG-CMV vector (Sigma, USA), respectively. The pEF1/myc-His-Flot-1 and pFLAG-CMV-Flot-2 vectors were transfected into 293T cells in different combinations. Forty-eight hours later, cells lysates were prepared and pre-incubated with agarose IgA/IgG beads for 2 h at 4 °C. Then, beads were removed and fresh agarose IgA/IgG beads with anti-His or anti-Flag were incubated with the lysates overnight at 4 °C. After washing, denaturation, and SDS-PAGE, the proteins were visualized by immunoblotting. The endogenous interactions in 5-8F cells were analyzed in a similar way with anti-Flot-1. The experiment was repeated for three independent times.

### Colony formation assay and soft agar assay

Colony formation assays were performed in accordance with a published manual^50^ , and soft agar assays were performed under standard assay conditions^51^. Each assay was performed in triplicate independently. The data are expressed as the means±SD of the number of colonies. The experiments were repeated for three independent times.

### *In vitro* cell proliferation assay

MTT assays were performed to assess the effect of Flot-2 on cell proliferation according to a published protocol^52^. The experiment was repeated for three independent times.

### Fluorescence-activated cell sorting (FACS)

FACS analysis was carried out as described previously^53^. The experiment was repeated for three independent times.

### *In vitro* Matrigel invasion assay

This assay was based on the principle of the Boyden chamber. Cells (5 × 10^4^) were suspended in serum-free medium and loaded into the upper compartment of invasion chambers coated with Matrigel (BD Biosciences). The lower compartments were filled with medium. After 48 h, invasive cells were fixed, stained, and counted in five predetermined fields under a microscope. The data are expressed as the average number of cells migrating through the filters. The experiment were repeated for three independent times.

### Migration assay and *in vitro* scratch wound healing assay

The procedures for the migration assay were similar to those described for the Matrigel invasion assay except that no Matrigel was used and the incubation time was 16 h. Scratch wound healing assays were conducted according to our published protocol^53^. The experiments were repeated for three independent times.

### Detection of distant metastases in nude mice

Cells (2 × 10^6^) from each cell line were injected intraperitoneally into groups of five 5-week-old BALB/C^−nu/nu^ mice (SLAC Laboratory Animal Co., Shanghai, China). Mice were sacrificed 5 weeks later. All mice were carefully checked by routine biopsy. The lungs and mediastinal lymph nodes were removed and examined by H&E staining. All animal procedures were approved by the Animal Care and Use Committee of Central South University and performed in accordance with institutional policies.

### Cytoskeleton observation

Cells were first stained with phalloidin-TRITC (50 μg/ml, Sigma, USA). The cellular morphological changes were observed under a light microscope (TE2000U, Nikon, Japan) and cytoskeleton visualization was recorded with a laser scanning confocal fluorescence microscope (Carl Zeiss Inc., Germany).

### Statistical analysis

A Kruskal-Wallis H test was performed to compare the difference in Flot-2 expression among NP (a), non-metastatic (b) and metastatic NPC (c) groups, and the Nemenyi test was then further used to perform pairwise comparisons among a, b and c groups. Differences between mean values were assessed by a two-tailed student *t*-test. For all analyses, SPSS 13.0 statistical software (SPSS, Chicago, IL) was used. A value of *P* < 0.05 was regarded as statistically significant.

## Additional Information

**How to cite this article**: Liu, J. *et al.* Flotillin-2 promotes metastasis of nasopharyngeal carcinoma by activating NF-κB and PI3K/Akt3 signaling pathways. *Sci. Rep.* **5**, 11614; doi: 10.1038/srep11614 (2015).

## Supplementary Material

Supplementary Information

## Figures and Tables

**Table 1:** Comparison of Flot-2 expression in NP and NPC tissues.

**Characteristics**	**Flotillin-2 expression**					
	**Negative**	**Weak**	**Moderate**	**Strong**	**Total**	***P* value**
	(0)	(1–4)	(5–9)	(10–15)		
NP (a)	30	8	0	0	38	*P*_a-b_ < 0.01
Non-metastatic NPC (b)	0	19	19	7	45	*P*_b-c_ < 0.05
Metastatic NPC (c)	0	15	38	34	87	*P*_a-c_ < 0.01

*P*_a-b_: probability value for Flot-2 expression difference between NP and non-metastatic NPC. *P*_b-c_: probability value for Flot-2 expression difference between non-metastatic NPC and metastatic NPC. *P*_a-c_: probability value for Flot-2 expression difference between NP and metastatic NPC.
