# Author Correction: Flotillin-2 promotes metastasis of nasopharyngeal carcinoma by activating NF-κB and PI3K/Akt3 signaling pathways

**DOI:** 10.1038/s41598-020-63750-w

**Published:** 2020-04-20

**Authors:** Jie Liu, Wei Huang, Caiping Ren, Qiuyuan Wen, Weidong Liu, Xuyu Yang, Lei Wang, Bin Zhu, Liang Zeng, Xiangling Feng, Chang Zhang, Huan Chen, Wei Jia, Lihua Zhang, Xiaomeng Xia, Yuxiang Chen

**Affiliations:** 1 Cancer Research Institute, Collaborative Innovation Center for Cancer Medicine, Key Laboratory for Carcinogenesis of Chinese Ministry of Health, School of Basic Medical Science, Central South University, Xiangya Road 110, 410078 Changsha, Hunan P. R. China; 2 Department of Pathology, Hunan Cancer Hospital, Changsha, Hunan P. R. China; 3 Department of Gynaecology and Obstetrics, The Second Xiangya Hospital, Central South University, Changsha, Hunan P. R. China; 4 Hepatobiliary & Enteric Surgery Research Center, Xiangya Hospital, Central South University, Changsha, Hunan P. R. China

Correction to: *Scientific Reports* 10.1038/srep11614, published online 24 July 2015

This Article contains an error. The incorrect anti-His blot, for immunoprecipitation with Flag Ab, was used in Figure 8A. The correct version of this panel appears below, as Figure 1.

**Figure 1:**
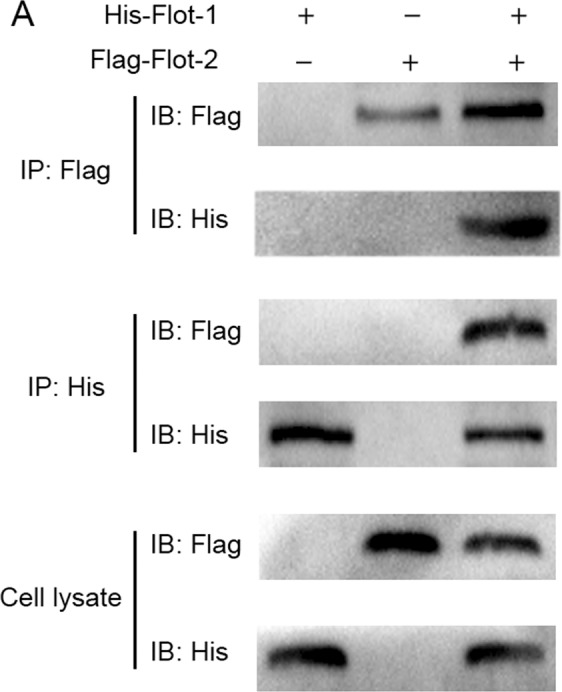
.

The original uncropped blots for figure 8A appear below, as Figure 2.

**Figure 2:**
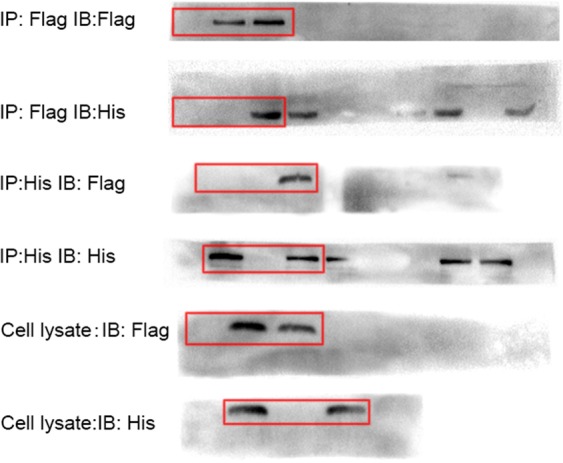
.

