# Author Correction: Flotillin-2 promotes metastasis of nasopharyngeal carcinoma by activating NF-κB and PI3K/Akt3 signaling pathways

**DOI:** 10.1038/s41598-023-39544-1

**Published:** 2023-08-01

**Authors:** Jie Liu, Wei Huang, Caiping Ren, Qiuyuan Wen, Weidong Liu, Xuyu Yang, Lei Wang, Bin Zhu, Liang Zeng, Xiangling Feng, Chang Zhang, Huan Chen, Wei Jia, Lihua Zhang, Xiaomeng Xia, Yuxiang Chen

**Affiliations:** 1 Cancer Research Institute, Collaborative Innovation Center for Cancer Medicine, Key Laboratory for Carcinogenesis of Chinese Ministry of Health, School of Basic Medical Science, Central South University, Xiangya Road 110, Changsha, 410078 Hunan People’s Republic of China; 2 Department of Pathology, Hunan Cancer Hospital, Changsha, Hunan People’s Republic of China; 3 Department of Gynaecology and Obstetrics, The Second Xiangya Hospital, Central South University, Changsha, Hunan People’s Republic of China; 4 Hepatobiliary and Enteric Surgery Research Center, Xiangya Hospital, Central South University, Changsha, Hunan People’s Republic of China

Correction to: *Scientific Reports* 10.1038/srep11614, published online 24 July 2015

This Article contains an error.

As a result of an error during figure assembly, in Figure 2A the image for the loading control for HONE1/HK1/C666-1 is a duplication of the image for the loading control for HNE1/HNE2/HNE3.

The correct Figure 2 is shown below, as Figure 1.

**Figure 1:**
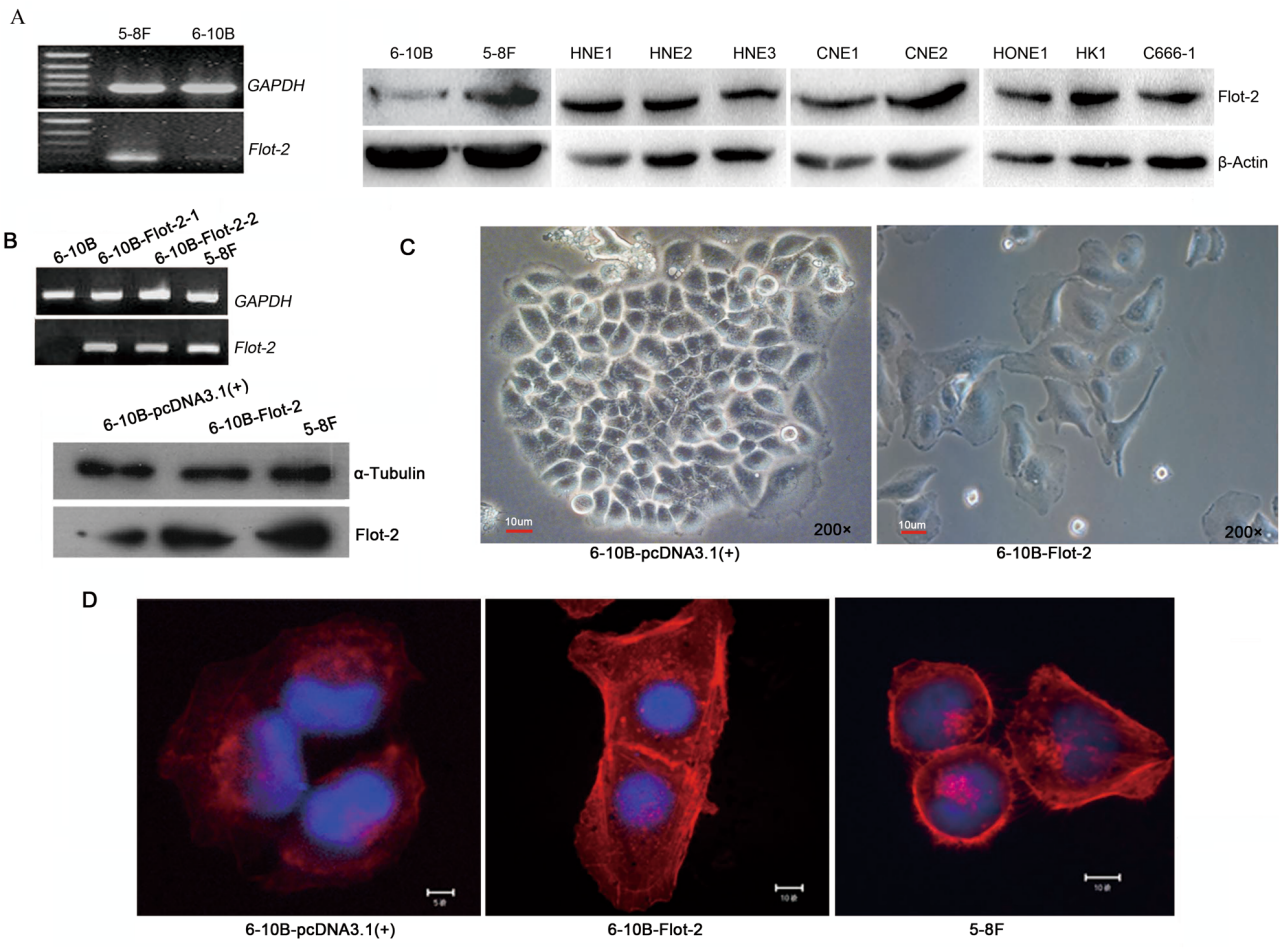
The effect of Flot-2 overexpression on the morphology of 6-10B cells. (**A**) The Flot-2 expression level in 5-8F, 6-10B and other NPC cells was detected by semi-quantitative RT-PCR and Western blotting. The expression of Flot-2 in 6-10B was weaker than that in other NPC cells. (**B**) Semi-quantitative RT-PCR and Western blotting were used to detect Flot-2 expression in 6-10B-Flot-2 cells. The 6-10B-Flot-2 cells achieved a comparable Flot-2 expression level to that in 5-8F cells. (**C**) The morphology of 6-10B and 6-10B-Flot-2 cells observed by inverted microscopy (200×). 6-10B-Flot-2 cells had a mesenchymal-like morphology with lamellipodia. (**D**) Cytoskeleton of 6-10B, 6-10B-Flot-2 cells and 5-8F cells were recorded under confocal laser-scanning microscope. 6-10B-Flot-2 cells exhibited a similar microfilament distribution pattern to 5-8F cells, consisting of a high-density distribution of microfilaments on the cell surface, which precedes the formation of conspicuous lamellipodia and membrane ruffles.

