# Supplementary material for: Flotillin-2 promotes metastasis of nasopharyngeal carcinoma by activating NF-κB and PI3K/Akt3 signaling pathways
Source: Sci Rep. 2015 Jul 24;5:11614. doi: 10.1038/srep11614 (PMC4648439; doi:10.1038/srep11614)
Supplement: Supplementary Information [file srep11614-s1.pdf]

Flotillin-2 promotes metastasis in nasopharyngeal carcinoma through activating NF-kB and PI3K/Akt3 signaling pathways

Jie Liu[1†], Wei Huang [1†], Caiping Ren [1]*, Qiuyuan Wen [1], Weidong Liu [1], Xuyu Yang [1], Lei Wang [1], Bin Zhu [1], Liang Zeng [2], Xiangling Feng [1], Chang Zhang [1], Huan Chen[1], Wei Jia[1], Lihua Zhang[1], Xiaomeng Xia[3], Yuxiang Chen [4]*

[†]Equal contributors

1Cancer Research Institute, Collaborative Innovation Center for Cancer Medicine, Key Laboratory for Carcinogenesis of Chinese Ministry of Health, Central South University, Xiangya Road 110, 410078, Changsha, Hunan, P. R. China. 2Department of Pathology, Hunan Cancer Hospital, Changsha, Hunan,c. 3Department of gynaecology and obstetrics , The Second Xiangya Hospital, Central South University, Changsha, Hunan, P. R. China. 4Hepatobiliary & Enteric Surgery Research Center, Xiangya Hospital, Central South University, Changsha, Hunan, P. R. China.

* Correspondence: Dr Caiping Ren, rencaiping@csu.edu.cn and Dr Yuxiang Chen, chenyx008@aliyun.com

Suppleplementry Figure 1 Checking the expressions of randomly selecetive genes from microarray by qPCR.

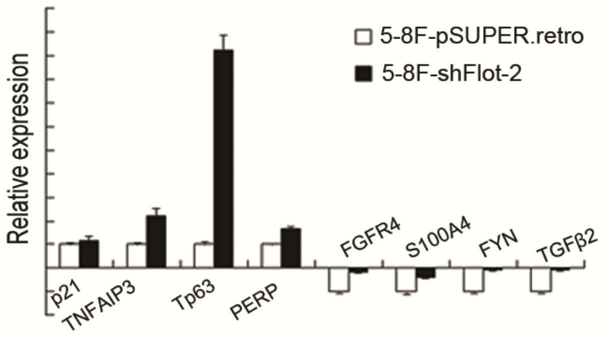

Suppleplementry Figure 2 Original western blot pictures for p-Akt3(S472). The square frames stand for the clipped regions.

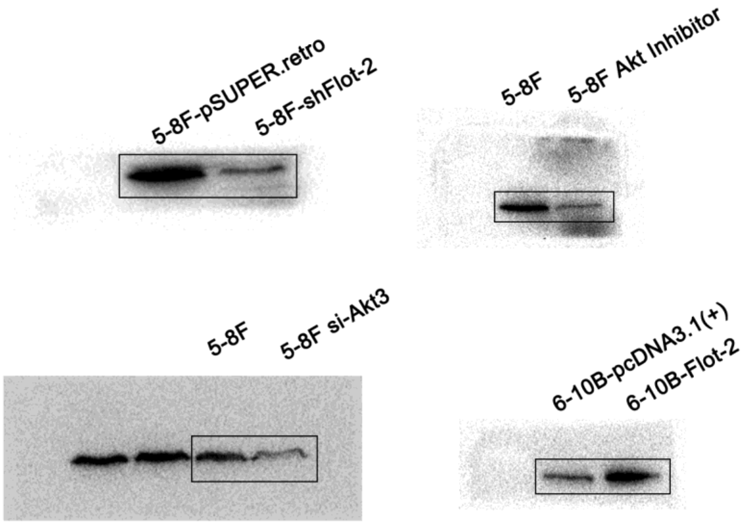

Suppleplementry Figure 3 Original western blot pictures for NF-κ B factors . The square frames stand for the clipped regions.

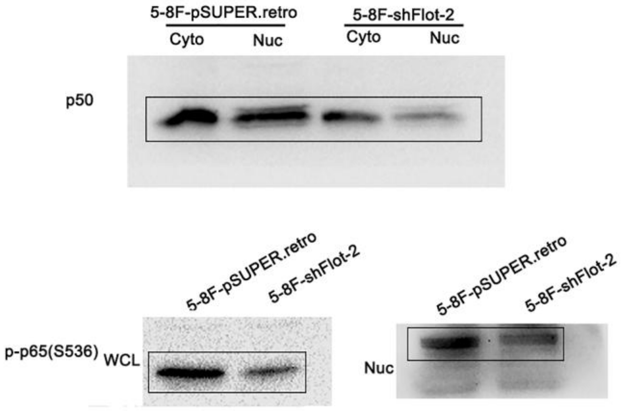

Suppleplementry Figure 4 Original western blot pictures for p-Foxo1(S256). The square frames stand for the clipped regions.

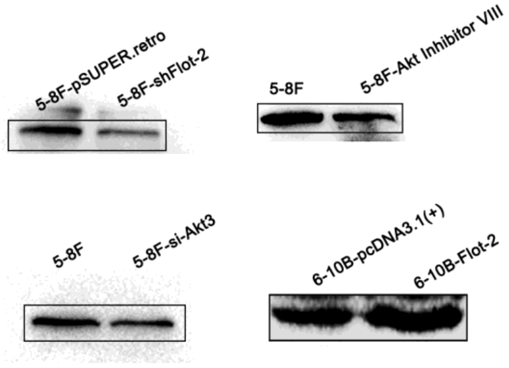

Suppleplementry Figure 5 Original western blot pictures for E-cadherin and MMP2-9. The square frames stand for the clipped regions.

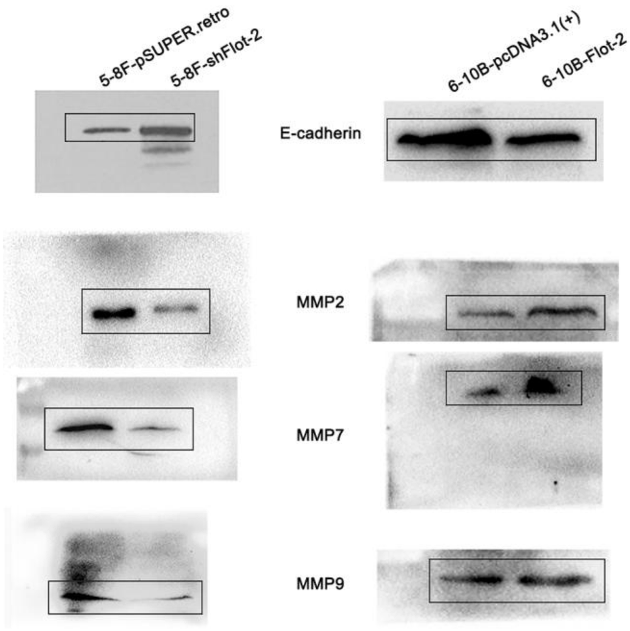

Supplementry table 1: Some down-regulated and up-regulated genes after knockdown Flot-2 in 5-8F cells

GeneBank	Symbol	Description	Fold Change
Down-regulated			
NM_002011.3	FGFR4	Fibroblast Growth Factor 4	0.4352
NM_002961.2	S100A4	S100 Calcium-Binding Protein A4	0.3329
NM_002037.5	FYN	OKT3-Induced Calcium Influx Regulator	0.2623
NM_001135599.2	TGFB2	ransforming Growth Factor, Beta 2	0.3712
NM_001251825.1	SP1	Sp1 Transcription Factor	0.4717
NM_001111045.1	CCNA1	Cyclin A1	0.1065
NM_057749.2	CCNE2	G1/S-Specific Cyclin-E2	0.3869
NM_006500.2	MCAM	Melanoma Cell Adhesion Molecule	0.3452
Up-regulated			
NM_000389.4	p21	Cyclin-Dependent Kinase Inhibitor 1A	2.0928
NM_001270507.1	TNFAIP3	Tumor Necrosis Factor, Alpha-Induced Protein 3	7.3011
NM_001114978.1	TP63	Tumor Protein P63	11.6372
NM_022121.4	PERP	TP53 Apoptosis Effector	3.8793
NM_001206696.1	IRF6	Interferon Regulatory Factor 6	8.5355
NM_000043.4	FAS	Fas Cell Surface Death Receptor	2.0733
NM_002178.2	IGFBP6	Insulin-Like Growth Factor Binding Protein 6	2.4663
NM_001257135.1	LIF	Leukemia Inhibitory Factor	2.4335

Supplementary Table 2: Primers for qPCR

Symbol	Primer Sequences	Product length
S100A4	Forward primer:TTCCCCTCTCTACAACCCTCT<br>Reverse primer:ACAGCAGTCAGGATCAAACCA	64bp
FYN	Forward primer:CCTTTCTTATCCGCGAGAGTGA<br>Reverse primer:GGTCTCCTTTCATATCATCCCAAT	81bp
TGFβ2	Forward primer:CAGCACACTCGATATGGACCA<br>Reverse primer:CCTCGGGCTCAGGATAGTCT	113bp
CCNA1	Forward primer:GAGGTCCCGATGCTTGTCAG<br>Reverse primer:GTTAGCAGCCCTAGCACTGTC	82bp
MCAM	Forward primer:TGGTTTGTACACCTTGCAGAGTATTC<br>Reverse primer:TGGGCAGCCGGTAGTTGA	95bp
FGFR4	Forward primer:GAGGGGCCGCCTAGAGATT<br>Reverse primer:CAGGACGATCATGGAGCCT	85bp
CCNE2	Forward primer:TCAAGACGAAGTAGCCGTTTAC<br>Reverse primer:TGACATCCTGGGTAGTTTTCCTC	115bp
Sp1	Forward primer:ACCACTACCATTTCCATTGC<br>Reverse primer:ACTCCATGGATGAAATGACA	83bp
CDKN1A	Forward primer:CCTCATCCCGTGTTCTCCTTT<br>Reverse primer:GTACCACCCAGCGGACAAGT	97bp
TP63	Forward primer:GTCATTTGATTCGAGTAGAGGGG<br>Reverse primer:CTGGGGTGGCTCATAAGGT	98bp
TNFAIP3	Forward primer:TCCTCAGGCTTTGTATTTGAGC<br>Reverse primer:TGTGTATCGGTGCATGGTTTTA	124pb
PERP	Forward primer:CTTCACCCTTCATGCCAACC<br>Reverse primer:GCCAATCAGGATAATCGTGGCT	88bp
GAPDH	Forward primer:CCAGCAAGAGCACAAGAGGAA<br>Reverse primer:ATGGTACATGACAAGGTGCGG	84bp
